# Association Between Diastasis of the Rectus Abdominis Muscles and Musculoskeletal Conditions in the First 2 Years Postpartum: A Cross‐Sectional Study

**DOI:** 10.1002/msc.70209

**Published:** 2026-03-23

**Authors:** Eloise Simpson, Madeline Hannington, Kari BØ, Andrew Hahne

**Affiliations:** ^1^ Physiotherapy Department South West Healthcare Warrnambool Victoria Australia; ^2^ Discipline of Physiotherapy School of Allied Health, Human Services & Sport La Trobe University Bundoora Victoria Australia; ^3^ Department of Sports Medicine Norwegian School of Sport Sciences Oslo Norway

**Keywords:** abdominal pain, diastasis rectus abdominis, low back pain, pelvic floor dysfunction, pelvic girdle pain, physiotherapy

## Abstract

**Objective:**

To explore associations between the presence and severity of diastasis of the rectus abdominis muscles (DRAM) and common postpartum musculoskeletal complaints in the first 2 years postpartum.

**Study Design:**

Cross‐sectional survey among women within 2 years of childbirth.

**Background:**

DRAM commonly affects women during pregnancy and postpartum, but its relationship with musculoskeletal complaints remains unclear. This study investigates the association between DRAM and postpartum musculoskeletal complaints, including low back pain, pelvic girdle pain, abdominal pain, and pelvic floor dysfunction.

**Methods and Measures:**

Participants were identified from medical records of women who had delivered a baby in the previous 2 years at Southwest Healthcare, Warrnambool, Victoria, Australia. An electronic questionnaire collected data on DRAM presence and severity (self‐reported based on self‐assessment or prior healthcare professional screening), musculoskeletal complaints, and pelvic floor dysfunction both currently (within the past week) and early postpartum (within the first 3 months). Statistical univariate and multivariate regression analyses explored associations between DRAM presence or severity and reported symptoms adjusted for age, parity, delivery method and time since last delivery.

**Results:**

Of 177 respondents (from 785 survey invitations), 38% (*n* = 70) reported DRAM. In multivariate analysis, DRAM presence was significantly associated with current (*p* = 0.034) and early postpartum (*p* = 0.037) abdominal discomfort, and urinary urgency symptoms early postpartum (*p* = 0.033). No significant associations were found between DRAM and low back pain, pelvic girdle pain, or stress urinary incontinence.

**Conclusion:**

DRAM was weakly associated with abdominal discomfort and urinary urgency symptoms but not with other musculoskeletal complaints. These findings align with limited previous research on this topic. More data are needed to explore the association between DRAM severity and musculoskeletal disorders.

## 1 Introduction

Diastasis of the rectus abdominis muscles (DRAM) is a condition where the rectus abdominis (RA) muscle bellies separate due to thinning and widening of the linea alba without defect of the fascia (Cavalli et al. 2021). Although DRAM may affect men and women, it is highly prevalent during the third trimester of pregnancy and after childbirth (Nienhuijs et al. 2021; Sperstad et al. 2016). The presence of DRAM and subsequent severity is typically determined by measuring the distance between the two RA muscle bellies at various points along the linea alba, which is referred to as the inter‐recti distance (IRD) (Benjamin et al. 2020).

There is currently no universally accepted definition of what classifies as DRAM, and different normative values have been reported across populations (Djivoh et al. 2022; Reinpold et al. 2019; Tuominen et al. 2022). Variations in methods of measurement (primarily finger width, calipers or ultrasound), testing position (crook lie, supine lie, at rest or with head lift), inconsistent measurement points along the linea alba, and limited consensus on diagnostic criteria and treatment, may have contributed to poor identification and understanding of the condition across both postpartum women and healthcare professionals (Benjamin et al. 2020; Gustavsson and Eriksson‐Crommert 2020; Water and Benjamin 2016; Weingerl et al. 2023).

There is limited and conflicting evidence on the musculoskeletal implications of DRAM (Benjamin et al. 2019). DRAM may be associated with more severe low back pain, but evidence is inconsistent regarding the relationship between DRAM and the overall incidence of low back pain. There have been inconsistent findings in studies examining the relationship between DRAM and pelvic girdle pain, although stabilisation exercises in the postpartum period have been found to reduce pelvic girdle pain in the postpartum period (Özyurt et al. 2025). Previous research has also found that DRAM may be associated with the presence of abdominal pain and decreased abdominal muscle strength (S. B. Gluppe et al. 2023; Keshwani et al. 2018).

The relationship between abdominal muscle strength and DRAM has been explored in further research (S. Gluppe et al. 2021a; Starzec‐Proserpio, Rejano‐Campo, et al. 2022). Postpartum women with pelvic girdle pain were found to be more likely to have increased inter‐recti distance (IRD) during a curl up task compared with pain‐free controls (Starzec‐Proserpio, Lipa, et al. 2022). One study found participants who achieved a greater reduction in inter‐recti distance following completion of upper abdominal exercises also had a concurrent reduction in low back pain severity (Saleem et al. 2021). However, there have also been contrary findings to each of the above associations (Baran et al. 2021; Benjamin et al. 2019; Doubkova et al. 2018; Fei et al. 2021; Gitta et al. 2017; S. Gluppe et al. 2021a; Joueidi et al. 2019; Tian et al. 2021). The heterogeneity of measurement methods combined with high risk of bias in existing research studies may have clouded understanding of the potential sequelae of DRAM (Benjamin et al. 2023; Weingerl et al. 2023).

There is limited research evidence on how musculoskeletal complaints may vary with different severities of DRAM (mild < 3 cm, moderate 3–5 cm, or severe > 5 cm) (Reinpold et al. 2019). Although severe DRAM has previously been found to be rare, it tends to be more persistent. Previous research has found that 41% of severe DRAM cases are still detectable at 3–6 months postpartum, and 32.6% at 12 months after childbirth (Cardaillac et al. 2020). However, most current research has focused on mild to moderate DRAM, and little is known about the impacts of severe DRAM. It is therefore not known whether the presence or severity of musculoskeletal dysfunction varies with DRAM severity (S. Gluppe et al. 2021b).

Despite these uncertainties, several theoretical mechanisms have been proposed. DRAM may plausibly influence musculoskeletal and pelvic floor symptoms through altered abdominal wall tension, potentially compromised force transfer across the anterior abdominal wall, and consequential changes in intra‐abdominal pressure regulation (Benjamin et al. 2019). Given the close working relationship between the abdominal musculature and pelvic floor during load transfer and postural tasks, changes to integrity of the abdominal wall may theoretically contribute to the musculoskeletal or pelvic floor dysfunctions explored in this manuscript (Sapsford and Hodges 2001; Sapsford et al. 2001).

The aim of this study was to explore associations between the presence and severity of DRAM and common postpartum musculoskeletal complaints (low back pain, pelvic girdle pain, pelvic floor dysfunction and abdominal pain) during the first 2 years postpartum.

## 2 Methods and Measures

### 2.1 Research Design

This was a descriptive cross‐sectional study conducted via an online survey.

### 2.2 Participants

Participants who had given birth at Southwest Healthcare's primary campus were invited to participate in this study. This is a public hospital in the major regional town of Warrnambool, Australia, and contains the only maternity ward in the region, capturing all public and privately insured mothers who choose to birth in a hospital.

Participants were eligible for inclusion if they had given birth via any mode of delivery between 2 weeks and 2 years from the opening date of the survey. For sensitivity reasons, participants did not receive an invitation to participate if they had experienced stillbirth or infant death within the first 2 years following birth. Mothers under 18 years of age at the time of giving birth were also excluded. There was no limit placed on the number of infants delivered during the pregnancy.

A personalised survey invitation link was sent via email through the programme REDCap for participants to complete on their personal electronic device (Harris et al. 2019, 2009). A reminder email was sent 2 weeks following the initial invitation.

### 2.3 Ethical Consideration

This study was approved by the South West Healthcare Ethics Committee (LNR/90220/SWH‐2023‐352408 (v2)). Informed consent was obtained at the commencement of the survey prior to data collection. The survey was anonymous. The 136‐question instrument took between 5 and 12 min to complete depending on participant responses. Less time was required if the respondent reported not having DRAM or symptoms of musculoskeletal pain or pelvic floor dysfunction.

### 2.4 Questionnaires

The authors developed the survey tool using a range of questions adapted from validated questionnaires, yes/no response questions and illustrations (Baessler et al. 2010; S. Gluppe et al. 2021a; Peterson et al. 2010). The survey was composed of three main sections.

#### 2.4.1 General Participant Demographics

Data collected included participants' age bracket, parity, education level, Aboriginal or Torres Strait Islander heritage, length of time since their most recent birth and the number of infants born at that time.

#### 2.4.2 Presence and Severity of DRAM

**FIGURE 1:**
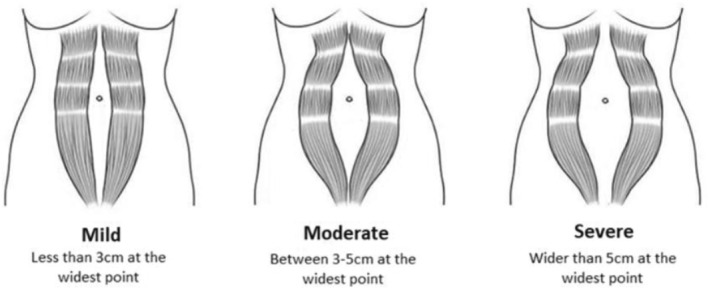
Illustration used as a multiple‐choice option in the survey to establish DRAM severity.

Participants were asked to identify the presence of DRAM at any time since their most recent birth as identified by themselves or a health professional. Although limited data on the validity of self‐assessment of DRAM exists, self‐assessment has previously been found to be a reliable measure of DRAM after a session with a clinician (Cardaillac et al. 2020). Using Figure 1 as a guide, participants reporting DRAM were asked to select the image which most closely resembled their own DRAM at its widest time. Options correlated with ‘mild,’ ‘moderate’ or ‘severe’ from left to right based on the proposed international classification (Reinpold et al. 2019). This approach was used to capture perceived presence and severity of DRAM in a population‐based sample; it does not quantify inter‐recti distance and should be interpreted accordingly. Participants reporting DRAM were also asked ‘how much did/does your DRAM bother you?’ using an 11‐point scale (0 = not at all bothered, 10 = Extremely bothered). Potential predictive factors of DRAM such as parity and demographic data were also collected.

#### 2.4.3 Common Postpartum Musculoskeletal Complaints

Questions specific to low back pain, pelvic girdle pain, abdominal pain or discomfort and pelvic floor dysfunction were asked of all participants. These questions were asked in relation to two distinct time periods: ‘early postpartum’ (within the first 3 months after giving birth) and ‘current’ (within the past week). The abdominal pain section was adapted from a previous study investigating abdominal pain and/or discomfort in postpartum women with DRAM (S. Gluppe et al. 2021a). The pelvic floor dysfunction section was adapted from the pelvic floor screening tool for women developed by the Continence Foundation of Australia as part of the Pelvic Floor First campaign (Baessler et al. 2010). Each of the common postpartum musculoskeletal complaint sections including pelvic girdle pain, abdominal pain, low back pain and associated radiation of symptoms was accompanied by the relevant section of a body chart with the area of interest shaded to improve ease of understanding of each condition.

### 2.5 Data Analysis

All statistical analyses were performed using SPSS statistical software (Version 29.0.0). Participant demographics and descriptive survey data were reported as frequencies and percentages. For descriptive purposes, we examined the association between higher parity and the presence/severity of DRAM, and between DRAM severity and DRAM‐related bother. For the primary analysis, participants with and without DRAM were compared for musculoskeletal pain and pelvic floor dysfunction outcomes. A two‐stage process was used to minimise the risk of inflated Type 1 errors, with potential candidate variables identified via univariate analysis and then progressing to multivariate analysis. Conclusions were drawn only from multivariate models. For continuous variables, Pearson's r bivariate correlation coefficient was used to calculate the strength of individual associations. Categorical variables were calculated using Pearson's chi‐square test of association. Significance was set at *p* = 0.05.

A binary logistic regression analysis was then conducted for all significant univariate findings to determine if the presence of DRAM—coded as present (0) or not present (1)—remained a significant factor after controlling for potential confounders. This included adjusting for age, delivery method (0 = vaginal, 1 = Caesarean section), parity (continuous variable) and time since most recent delivery (continuous). The same process was repeated for the severity analysis, where the incidence of musculoskeletal conditions was compared in women with severe DRAM relative to those with mild/moderate DRAM using univariate and then multivariate analyses (if applicable).

## 3 Results

**FIGURE 2:**
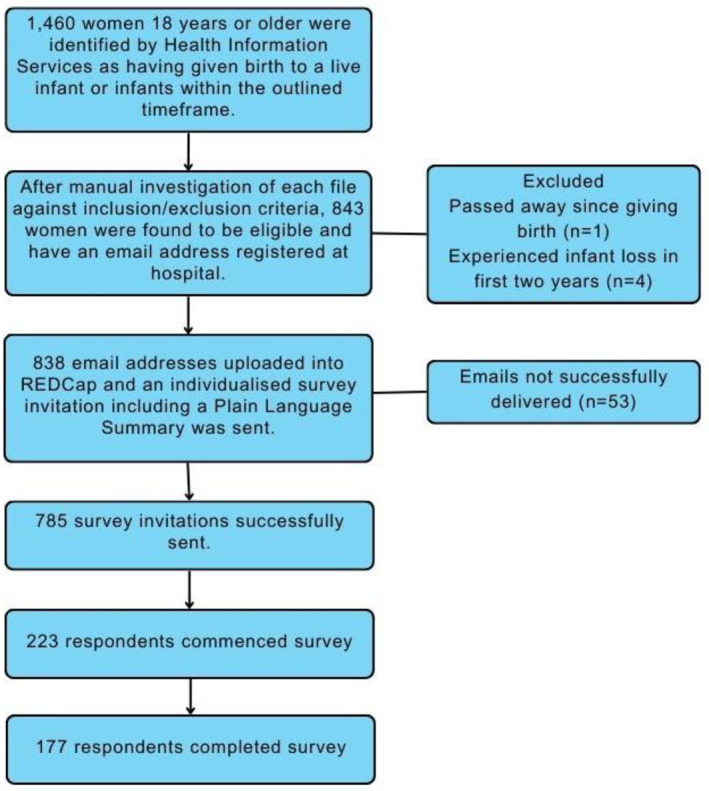
Participant flow.

Participant flow through the study is detailed in Figure 2. In total, 843 women were eligible for inclusion in this study. Emails were successfully sent to 785 participants, with 177 participants completing the survey (23% response rate) (Figure 2). Most participants were aged between 28 and 32 years (Table 1), with the mean time since giving birth 12.5 months. The participants had a mean of 1.9 pregnancies to 20 weeks of gestation or more. Univariate associations between DRAM and musculoskeletal conditions or pelvic floor dysfunction are presented in Table 2. Table 3 shows the results of multivariate binary logistic regression adjusting for age, parity, delivery method and time since the most recent delivery.

**TABLE 1:** Participant demographics.

Measure	Items	Frequency (*n*)	Percentage (%)
Age	18–22 years	5	2.3
	23–27 years	31	14.0
	28–32 years	89	40.3
	33–37 years	64	29.0
	38–42 years	31	14.0
	43 years +	1	0.4
Parity	1	88	39.8
	2	81	36.7
	3	40	18.1
	4	10	4.5
	5+	2	0.9
Number of infants born at last birth	1—singleton	216	97.7
	2—twins	5	2.3

**TABLE 2:** Presence of musculoskeletal dysfunction in those with and without DRAM.

	No DRAM *n*(%)	DRAM (any) *n*(%)	DRAM (mild) *n*(%)	DRAM (moderate) *n*(%)	DRAM (severe) *n*(%)
Low back pain					
Low back pain +/− leg pain in first 3 months postpartum	42(60.9)	40(66.7)	22(62.9)	17(65.4)	1(20.0)
Low back pain +/− leg pain lasting at least 24 h in the past week	27(39.1)	24(36.4)	13(37.1)	11(42.3)	0(0.0)
Referred leg pain in the past week	7(25)	14(31.8)	8(61.5)	6(54.4)	N/A
Pelvic girdle pain					
Pelvic girdle pain in the first 3 months postpartum	31(44.9)	23(66)	9(25.7)	14(53.8)	0(0.0)
Pelvic girdle syndrome in the first 3 months postpartum	8(25.8)	9(39.1)	4(44.4)	5(35.7)	N/A
Pelvic girdle pain lasting at least 24 h in the past week	7(10.1)	13(19.7)	4(11.4)	9(34.6)	0(0.0)
Pelvic girdle pain in 2 or more locations in the last week	2(28.6)	7(53.8)	4(100.0)	3(33.3)	N/A
Abdominal pain					
Abdominal pain or discomfort in the first 3 months postpartum	33(47.8)	40(61.5)	20(57.1)	15(60.0)	5(100.0)
Abdominal pain or discomfort in the past week	7(10.1)	13(20.0)	6(17.1)	6(24.0)	1(20.0)
Bladder and bowel function					
Stress urinary incontinence in the first 3 months postpartum	26(37.7)	31(44.3)	17(44.7)	10(37.0)	4(80.0)
Stress urinary incontinence in the past week	18(26.1)	18(25.7)	10(26.3)	6(22.2)	2(40.0)
No stress urinary incontinence	37(53.6)	21(30.0)	12(31.6)	9(33.3)	0(0.0)
Urgency +/− incontinence in the first 3 months postpartum	16(23.2)	26(37.1)	12(31.6)	10(37.0)	4(80.0)
Urgency +/− incontinence in the last week	10(14.5)	11(15.7)	6(15.8)	4(14.8)	1(20.0)
No urgency	48(69.6)	33(47.1)	20(52.6)	12(44.4)	1(20.0)
Difficulty emptying bladder+/−bowel in the first 3 months postpartum	12(17.4)	14(20.0)	7(18.4)	6(22.2)	1(20.0)
Difficulty emptying bladder+/−bowel in the last week	12(17.4)	9(12.9)	5(13.2)	4(14.8)	0(0.0)
No difficulty emptying bladder+/− bowel	45(65.2)	45(64.3)	25(65.8)	16(59.3)	4(80.0)
Bowel incontinence in the first 3 months postpartum	10(14.5)	14(20.0)	8(21.1)	4(14.8)	2(40.0)
Bowel incontinence in the last week	8(11.6)	4(5.7)	2(5.3)	1(3.7)	1(20.0)
No bowel incontinence	52(75.4)	48(68.6)	25(65.8)	21(77.8)	2(40.0)
Pelvic organ prolapse					
Symptoms of pelvic organ prolapse in the first 3 months postpartum	12(17.4)	16(22.9)	8(21.1)	7(25.9)	1(20.0)
Symptoms of pelvic organ prolapse in the last week	4(5.8)	5(7.1)	2(5.3)	2(7.4)	1(20.0)
No symptoms of pelvic organ prolapse	54(78.3)	47(67.1)	28(73.7)	16(59.3)	3(60.0)

**TABLE 3:** Results of binary logistic regression analysis including DRAM presence.

Outcome	*n*	Constant (SE), *p*; odds ratio	DRAM presence (SE), *p*; odds ratio [95% CI]	Age (SE), *p*; odds ratio [95% CI]	Delivery method (SE), *p*; odds ratio [95% CI]	Parity (SE), *p*; odds ratio [95% CI]	Time since birth (SE), *p*; odds ratio [95% CI]
Abdominal pain (first 3 months postpartum)	135	−1.45 (0.94), *p* = 0.125; 0.235	0.79 (0.38), *p* = 0.037; 2.209 [1.05–4.66]	0.41 (0.20), *p* = 0.043; 1.511 [1.01–2.25]	−0.47 (0.40), *p* = 0.234; 0.624 [0.29–1.36]	−0.05 (0.20), *p* = 0.800; 0.950 [0.64–1.41]	−0.06 (0.03), *p* = 0.020; 0.940 [0.89–0.99]
Abdominal pain in the past week	135	0.42 (1.28), *p* = 0.741; 1.526	1.14 (0.54), *p* = 0.034; 3.111 [1.09–8.90]	0.43 (0.28), *p* = 0.130; 1.537 [0.88–2.68]	−1.04 (0.51), *p* = 0.040; 0.353 [0.13–0.96]	−0.06 (0.27), *p* = 0.829; 0.943 [0.55–1.60]	−0.05 (0.04), *p* = 0.209; 0.955 [0.89–1.03]
Radiation of low back pain into lower limbs in the past week	52	−2.68 (1.88), *p* = 0.153; 0.068	−1.27 (0.73), *p* = 0.082; 0.281 [0.07–1.18]	0.10 (0.35), *p* = 0.978; 1.010 [0.51–2.02]	1.50 (0.72), *p* = 0.037; 4.498 [1.10–18.45]	0.07 (0.39), *p* = 0.854; 1.074 [0.50–2.30]	0.15 (0.06), *p* = 0.014; 1.161 [1.03–1.31]
Pelvic girdle syndrome in the first 3 months postpartum	55	1.94 (1.48), *p* = 0.189; 6.981	−1.07 (0.67), *p* = 0.107; 0.342 [0.09–1.26]	−0.31 (0.34), *p* = 0.370; 0.735 [0.38–1.44]	0.92 (0.70), *p* = 0.190; 2.518 [0.63–10.03]	−0.32 (0.41), *p* = 0.434; 0.726 [0.33–1.62]	−0.05 (0.05), *p* = 0.284; 0.952 [0.87–1.04]
Pelvic girdle syndrome in the past week	21	2.75 (4.03), *p* = 0.496; 15.572	−2.12 (1.59), *p* = 0.182; 0.120 [0.01–2.70]	−0.89 (0.91), *p* = 0.328; 0.410 [0.07–2.44]	−1.31 (1.37), *p* = 0.338; 0.271 [0.02–3.93]	−0.53 (0.70), *p* = 0.447; 0.588 [0.15–2.32]	0.16 (0.10), *p* = 0.119; 1.170 [0.960–1.43]
Stress urinary incontinence in the first 3 months postpartum	140	0.32 (0.93), *p* = 0.730; 1.377	−0.23 (0.37), *p* = 0.534; 0.796 [0.39–1.64]	0.04 (0.20), *p* = 0.858; 1.036 [0.70–1.53]	−1.00 (0.40), *p* = 0.013; 0.370 [0.17–0.81]	0.03 (0.20), *p* = 0.889; 0.972 [0.66–1.44]	−0.03 (0.03), *p* = 0.243; 0.971 [0.92–1.02]
Stress urinary incontinence in the past week	140	−1.05 (1.01), *p* = 0.300; 0.351	−0.10 (0.40), *p* = 0.805; 0.905 [0.41–1.99]	−0.13 (0.22), *p* = 0.563; 0.882 [0.58–1.35]	−0.29 (0.43), *p* = 0.506; 0.751 [0.32–1.74]	−0.05 (0.22), *p* = 0.809; 0.949 [0.61–1.46]	0.06 (0.03), *p* = 0.044; 1.059 [1.00–1.12]
Urge incontinence in the first 3 months postpartum	140	0.43 (0.98), *p* = 0.661; 1.537	−0.84 (0.39), *p* = 0.033; 0.432 [0.20–0.936]	−0.18 (0.21), *p* = 0.397; 0.835 [0.55–1.27]	−0.09 (0.41), 0.822; 0.912 [0.41–2.03]	−0.18 (0.21), *p* = 0.388; 0.833 [0.55–1.26]	0.01 (0.03), *p* = 0.672; 1.012 [0.96–1.07]
Urge incontinence in the past week	140	−2.24 (1.24), *p* = 0.072; 0.107	−0.20 (0.50), *p* = 0.692; 0.822 [0.31–2.17]	−0.06 (0.26), *p* = 0.819; 0.941 [0.56–1.58]	0.92 (0.49), *p* = 0.063; 2.496 [0.95–6.56]	−0.01 (0.27), *p* = 0.970; 0.990 [0.59–1.67]	0.04 (1.24), *p* = 0.072; 1.038 [0.97–1.11]

### 3.1 Assessment and Prevalence of DRAM

Over half the participants (50.5%, *n* = 93) reported being checked for DRAM following the birth of their most recent child by a healthcare professional, with 57% (*n* = 53) of those participants assessed in hospital prior to discharge. Women who reported being checked for DRAM prior to discharge were more likely to report the presence of DRAM (49.5%, *n* = 46) compared to those who did not recall being checked for DRAM (30.1%, *n* = 22) prior to discharge (*p* < 0.001). Women who did not recall being checked for DRAM prior to discharge home were also more likely to report being unsure about the presence of DRAM postnatally (35.6%, *n* = 26) compared to women who were screened before going home (7.5%, *n* = 7) (*p* < 0.001).

Overall, 38% (*n* = 70) of women reported the presence of DRAM in the postpartum period. Of these participants, 38 (54%) reported mild DRAM, 27 (39%) moderate DRAM and 5 (7%) reported the presence of severe DRAM at its widest point. There was a weak association between higher parity and the presence of DRAM (*r*(137) = 0.20, *p* = 0.020) but not severity (*r*(68) = 0.22, *p* = 0.062). A statistically significant relationship was found between higher DRAM severity and greater DRAM‐related bother, *r*(67) = 0.41, *p* < 0.001.

#### 3.1.1 Low Back Pain

There was no significant relationship between the presence of DRAM and low back pain currently (*φ* < 0.01, *p* = 0.975) or during early postpartum (*φ* = 0.03, *p* = 0.740). However, there was a significant univariate relationship between the presence of DRAM and current pain radiating to the lower limbs(s) from the back (*φ* = −0.329, *p* = 0.019). After controlling for age, parity, delivery method, and time since birth, the presence of DRAM was no longer an independent predictor of current low back pain with radiation of pain into the lower limbs (*p* = 0.051).

#### 3.1.2 Pelvic Girdle Pain

The presence of DRAM was associated with a higher number of pelvic pain sites in those reporting pelvic girdle pain, *r*(26) = 0.40, *p* = 0.038. However, there was no association between the presence of DRAM and the overall presence of pelvic girdle pain both currently and during early postpartum.

#### 3.1.3 Abdominal Pain

Univariate analysis showed a relationship between the presence of DRAM and current abdominal pain and/or discomfort, *V* = 0.26, df = 3, *p* = 0.028, and during early postpartum, *V* = 0.44, df = 6, *p* = 0.047. The presence of DRAM remained an independent predictor of both current and early postpartum abdominal pain in the multivariate analysis.

#### 3.1.4 Pelvic Floor Dysfunction

There was a significant univariate relationship between the reported presence of DRAM and symptoms of stress urinary incontinence (*r*(137) = 0.24, *p* = 0.005), and urinary urgency symptoms during early postpartum (*r*(137) = 0.23, *p* = 0.007). There was no association between the presence of DRAM and symptoms of pelvic organ prolapse (*r*(137) = 0.13, *p* = 0.143), difficulty emptying the bladder or bowel (*r*(137) = 0.01, *p* = 0.909) or incontinence of the bowel (r(137) = 0.08, *p* = 0.377). Following the binary logistic regression analysis, the presence of DRAM remained significantly associated with urinary urgency symptoms during early postpartum (*p* = 0.035) but was no longer associated with current urinary urgency symptoms (*p* = 0.105) or with stress urinary incontinence at either timepoint.

#### 3.1.5 DRAM Severity

Only 5 women in the sample reported severe DRAM; hence, the severity analysis was considered exploratory and statistically underpowered (Supporting Information S1: Appendix 1). Pearson's chi‐square test of association revealed no statistically significant association between DRAM severity and abdominal pain/discomfort (either currently or during early postpartum). Analysis of pelvic girdle syndrome was not possible because of missing data for all five severe DRAM cases. No definitive conclusions have been drawn from the severity analysis and these findings should be interpreted with caution.

## 4 Discussion

This study showed that the presence of self‐reported DRAM by postpartum women was not strongly associated with common musculoskeletal conditions in the first 2 years after childbirth. After adjusting for age, parity, time since birth and delivery method, the presence of DRAM may be associated with abdominal pain and/or discomfort, and symptoms of urinary urgency during the early postpartum period, but was not associated with low back pain, pelvic girdle pain, or symptoms of pelvic floor dysfunction such as stress urinary incontinence. Understanding the musculoskeletal sequelae of DRAM is important for prioritising its management, particularly if treatment is pursued to reduce dysfunction rather than for cosmetic reasons alone.

Pelvic girdle pain was not more prevalent in women with DRAM, but when present, it was reported across a higher number of pain sites with increased probability of participants reporting pelvic girdle syndrome in the presence of DRAM. A previous observational study also found no difference in postpartum rates of pelvic girdle pain between women with and without DRAM, but found that those with DRAM were more likely to experience disabling pelvic girdle pain antenatally (Zhu et al. 2024).

Given that the prognosis for pelvic girdle pain in the postpartum period is negatively correlated with the number of reported pain sites across the pelvic girdle joints (rather than with pain severity alone) (Wuytack et al. 2018), it is important to identify and address risk factors for developing pelvic girdle syndrome (defined as involvement of all three pelvic joints) (Wuytack et al. 2018). Further research should examine DRAM alongside other potential shared risk factors such as birth weight, activity levels, connective tissue integrity and body composition.

There was a weak but statistically significant association between DRAM presence and abdominal discomfort and/or pain both currently and during the early postpartum period. Previous research has found that abdominal pain was more prevalent (S. Gluppe et al. 2021a) and reported at a higher intensity (Parker et al. 2009) in postpartum women with DRAM than without. In the present study, abdominal pain during the early postpartum period was more frequently reported following caesarean section than vaginal birth, likely attributable to the surgical incision. Sixty‐seven percent of respondents who delivered via Caesarean Section had abdominal pain or discomfort in the early postpartum period compared with 50% of respondents who delivered vaginally. When asked about current abdominal pain and/or discomfort, there were still increased rates in the Caesarean section group (26% of respondents vs. 13% in the vaginal group). However, in this study, the presence of DRAM remained an independent predictor of abdominal pain at both timepoints when delivery method was controlled for.

The rates of pelvic floor dysfunction were similar in those with and without DRAM, although symptoms of urinary urgency were more prevalent in the population with DRAM. Changes in the abdominal wall could theoretically influence pelvic floor function (Sapsford 2001; Sapsford et al. 2001), confirmed by one EMG study showing decreased endurance of vaginal sphincter contraction in women with DRAM compared with controls (Zhang et al. 2023). However, many studies have found no difference in rates of pelvic floor dysfunction in women with versus without DRAM (Benjamin et al. 2019; Cardaillac et al. 2020; Fei et al. 2021; S. Gluppe et al. 2021a; Qu et al. 2021; Wang et al. 2020; Zhang et al. 2023). More research is needed to clarify the long‐term association between DRAM and different elements of pelvic floor function.

### 4.1 Strengths and Limitations

Strengths of this study include sampling from an entire population who delivered at the only hospital in the region, as well as the use of multivariate analysis to adjust for potential confounding factors that could account for musculoskeletal conditions.

A study limitation was the reliance on patient self‐report of DRAM, although this perspective had been verified by a healthcare practitioner check in 50% of the respondents. This does not eliminate the risk of incorrectly classified DRAM, especially as there was a higher DRAM prevalence in women who had been screened at the hospital versus those who were not, although this could be explained by women diagnosed with DRAM in hospital being more likely to remember the screening taking place.

The survey response rate of 23% introduces the possibility of non‐response bias, whereby respondents may not be fully representative of the population sampled.

Consistent with other literature, we found a very limited number of women reporting severe DRAM, so all findings related to severity were considered exploratory and underpowered. Further research involving women with severe DRAM would help clarify any relationships between DRAM severity and musculoskeletal conditions.

## 5 Conclusion

DRAM was not strongly associated with common musculoskeletal conditions in postpartum women in the first 2 years after childbirth. DRAM presence was significantly associated with current and early postpartum abdominal discomfort, and with early postpartum urgency urinary symptoms, but was not associated with low back pain, pelvic girdle pain, or symptoms of stress urinary incontinence. Large longitudinal studies that measure DRAM presence and severity during pregnancy and after birth and track outcomes at regular timepoints would help to aid understanding of the impacts of this condition on musculoskeletal and pelvic floor conditions.

## Author Contributions

Eloise Simpson conceived and designed the study, conducted data collection and statistical analysis, interpreted the results, and drafted the manuscript. Madeline Hannington contributed to study design, helped interpret statistical analysis and critically revised the manuscript. Kari BØ contributed to the conceptual framework and critically revised the manuscript. Andrew Hahne supervised the project, contributed to study design and analysis planning, and critically revised the manuscript. All authors approved the final manuscript.

## Conflicts of Interest

The authors declare no conflicts of interest.

## Supporting information


Supporting Information S1

## Data Availability

The data that support the findings of this study are available upon request from the corresponding author. The data are not publicly available due to privacy or ethical restrictions.
