# Supplementary material for: Association Between Diastasis of the Rectus Abdominis Muscles and Musculoskeletal Conditions in the First 2 Years Postpartum: A Cross‐Sectional Study
Source: Musculoskeletal Care. 2026 Mar 23;24(1):e70209. doi: 10.1002/msc.70209 (PMC13008823; doi:10.1002/msc.70209)
Supplement: Supplementary file 1 — Supporting Information S1 [file MSC-24-e70209-s001.docx]

**Appendix 1: DRAM severity analysis**

**Univariate analysis: Pearson Chi-Square test results for DRAM severity (mild/moderate vs severe)**

|  |  | **p=** |
| --- | --- | --- |
| **Pelvic girdle syndrome in the first 3 months** |  | **N/A** |
| **Pelvic girdle syndrome in the past week** |  | **N/A** |
| **Abdominal pain or discomfort in the first 3 months** |  | **.066** |
| **Abdominal pain or discomfort in the past week** |  | **1.00** |

N/A = could not compute due to pelvic girdle syndrome item missing for all severe DRAM cases.
